# Purification of Bioactive Peptide with Antimicrobial Properties Produced by *Saccharomyces cerevisiae*

**DOI:** 10.3390/foods9030324

**Published:** 2020-03-11

**Authors:** Shayma Thyab Gddoa Al-sahlany, Ammar B. Altemimi, Alaa Jabbar Abd Al-Manhel, Alaa Kareem Niamah, Naoufal Lakhssassi, Salam A. Ibrahim

**Affiliations:** 1 Department of Food Science, College of Agriculture, University of Basrah, Basrah 61004, Iraq; alsahlany.shayma@gmail.com (S.T.G.A.-s.); ammaragr@siu.edu (A.B.A.); alaafood_13@yahoo.com (A.J.A.A.-M.); 2 Department of Plant Soil and Agricultural Systems, Agriculture College, Southern Illinois University, Carbondale, IL 62901, USA; naoufal.lakhssassi@siu.edu; 3 Food and Nutritional Science Program, North Carolina A & T State University, Greensboro, NC 27411, USA; ibrah001@ncat.edu

**Keywords:** *Saccharomyces cerevisiae*, bioactive peptide, antibacterial, ÄKTA pure

## Abstract

A variety of organisms produce bioactive peptides that express inhibition activity against other organisms. *Saccharomyces cerevisiae* is considered the best example of a unicellular organism that is useful for studying peptide production. In this study, an antibacterial peptide was produced and isolated from *Saccharomyces* *cerevisiae* (Baker’s yeast) by an ultrafiltration process (two membranes with cut-offs of 2 and 10 kDa) and purified using the ÄKTA Pure 25 system. Antibacterial peptide activity was characterized and examined against four bacterial strains including Gram-positive and Gram-negative bacteria. The optimum condition for yeast growth and antibacterial peptide production against both *Escherichia. coli* and *Klebsiella aerogenes* was 25–30 °C within a 48 h period. The isolated peptide had a molecular weight of 9770 Da, was thermostable at 50–90 °C for 30 min, and tolerated a pH range of 5–7 at 4 °C and 25 °C during the first 24 h, making this isolated antibacterial peptides suitable for use in sterilization and thermal processes, which are very important aspect in food production. The isolated antibacterial peptide caused a rapid and steady decline in the number of viable cells from 2 to 2.3 log units of gram-negative strains and from 1.5 to 1.8 log units of gram-positive strains during 24 h of incubation. The isolated antibacterial peptide from *Saccharomyces cerevisiae* may present a potential biopreservative compound in the food industry exhibiting inhibition activity against gram-negative and gram-positive bacteria.

## 1. Introduction

*Saccharomyces cerevisiae* (typically known as baker’s yeast) is a single-cell eukaryote that is often utilized in research. *S. cerevisiae* has proven to be an ideal organism for research applications especially after releasing its genome sequence was released to the scientific community [1,2]. This yeast can be stored, and its genome sequence and translated proteins are similar in action to those of other organisms. Its proteome contains cell cycle and signaling proteins [3]. In the past few years, biologically active peptides have been produced in different sources such as food, plants, animals and microorganisms. Many studies have focused on the production, isolation, and purification of the antimicrobial peptides [4].

Antimicrobial peptides (AMPs) are small oligopeptides (AMPs) that typically contain 10–100 amino acids, with a net positive charge and an amphipathic structure [5]. Antimicrobial peptides have a broad spectrum of inhibition activity against microorganisms such as bacteria, molds, yeasts, parasites, and some viruses. Many living organisms such as microorganisms, invertebrates, and other species belonging to the animal and plant kingdoms produce antimicrobial peptides [6]. In addition, bioactive peptides produced during the fermentation process by different microorganisms have been used as antibacterial, antioxidant, antihypertensive, anticancer, and immunomodulatory agents, in addition to containing lipid-reducing properties [7]. Several studies have investigated the production of antimicrobial peptides from lactic acid, however, few studies focus on the production of antimicrobial peptides from yeasts in a medium [7,8].

In previous studies, metabolic compounds of *Saccharomyces boulardii* were separated and examined against 26 bacterial isolates in order to study the compounds’ antimicrobial activity. The extracted peptide from *Saccharomyces boulardii* has ben shown to exhibit high inhibition activity toward *Bacillus cereus* [9].

It has been reported that some *S. cerevisiae* strains have the ability to produce antimicrobial peptides. Recent studies demonstrated that the isolated *S. cerevisiae* CCMI 885 produced small peptides (<10 kDa) that exhibit antimicrobial activity against some yeasts such as *Hanseniaspora guilliermondii*, *Torulaspora delbrueckii*, *Kluyveromyces marxianus,* and *Lachancea thermotolerans* [10]. The ultrafiltration process with a 10 kDa cut-off was used to extract the antimicrobial peptide from *Candida intermedia* after growth on YPD agar at 28 °C over 7 days. The molecular weight of the peptide was 5 kDa exhibiting an inhibition activity against *Brettanomyces bruxellensis* [11].

Several authors have noted alternate strategies for biocontrol, such as the use of peptide-based killer toxins. For example, *Saccharomyces cerevisiae* produces peptide-based killer toxins that are able to inhibit the growth of different species of bacteria [12]. Similar results were reported for bioactive peptide production by *S. cerevisiae* CCMI 885 at 30 °C for 48 h. These peptides were used to inhibit the growth of some wine-related yeasts [8]. Past research including both in vitro and in vivo studies indicated that *Saccharomyces cerevisiae* inhibited intestinal tract infections from *Bacillus subtilis*, *B. cereus*, *Escherichia. coli*, *Proteus vulgaris*, *Pseudomonas aeruginosa*, *Salmonella typhimurium*, *Salmonella typhi*, *Staphylococcus aureus*, *Yersinia enterocolitica*, and some yeasts such as *Candida albicans* [13].

*Saccharomyces cerevisiae* yeast exhibits antibacterial activity against *E. coli*, *Pseudomonas* sp. *Salmonella* sp., *Staphylococcus aureus,* and *Vibrio cholera.* In addition, this yeast strain has antimicrobial activity against other pathogenic bacteria species, yeasts, and molds [14]. Many studies have described the effect of these antimicrobial activities on inhibition zones (zones free of growth).

To date, however, very limited information is available about bioactive peptides from *Saccharomyces cerevisiae*. Thus, the aim of this study was to investigate the antimicrobial components from a full extract of *Saccharomyces cerevisiae,* isolate antibacterial peptides, and test their thermostability characteristics which are very important aspect in food production including for sterilization and thermal processes.

## 2. Materials and Methods

### 2.1. Microbial Strains

*Saccharomyces cerevisiae* ATCC 36858 was obtained from a local scientific store in Basrah, Iraq. *Bacillus subtilis* ATCC 23857, *E. coli* ATCC 25922, *Klebsiella aerogenes* ATCC 13048, and *Staphylococcus aureus* ATCC 25923 were supplied by the Food Science Department/College of Agriculture/University of Basrah, Iraq and used as indicator strains.

### 2.2. Antibacterial Peptide Production

First, 1 mL (6 log cfu/mL) of activated yeast (36 h) was added to 250 mL of glucose yeast peptone broth (GTPB) medium (Himedia, Mumbai, India) and incubated at 20, 25, 30, and 35 °C for 24, 48, 72, and 96 h, respectively. After incubation, *S. cerevisiae* cells were removed by centrifugation at 6000 rpm for 20 min at 4 °C, and the cell free yeast supernatant was filtered with 0.45 µm cellulose acetate membranes (Merck company, Watford, UK) [9]. In order to isolate the peptides that were released from the yeast in the medium, the filtered metabolic yeast extract was passed through ultrafiltration membranes with pore sizes of 10 kDa (cut-off 10 kDa, Millipore and Amicon, USA) and then concentrated (100-fold) with 2 kDa (cut-off 2 kDa) membranes. The concentrated metabolic yeast extract was lyophilized by freeze-drying (Heto Lab Equipment, Denmark). Next, 100 mg/mL of lyophilized metabolic yeast was tested against four indicator strains (*Bacillus subtilis* ATCC 23857, *E. coli* ATCC 25922, *Klebsiella aerogenes,* and *Staphylococcus aureus* ATCC 25923) using the agar well diffusion agar method, and then 100 µL of lyophilized peptide was added to the wells (6 mm) in Nutrient agar (Himedia, Mumbai, India). After incubation, the clear zones were measured in millimeters [15,16].

### 2.3. Purification of the Antibacterial Peptide from Yeast

An ÄKTA Pure 25 System (GE Healthcare Life Sciences, Germany) was used to purify the antibacterial peptides from lyophilized metabolic extracts of *S. cerevisiae*. The specific column Superdex 200 (10/300GL) was used with a column volume set at 23.562 mL and a column diameter set at 10 mm with a pressure of 1.5 MPa. The column was filled with agarose and dextran. A 0.5 M acetate phosphate buffer (pH 5.0) at 0.5 mL/min was used for elution, and a 280 nm UV detector was used to determine the isolated peaks [17,18]. Peak fractions were collected, and the antibacterial activity against two indicator strains (*E. coli* ATCC 25922 and *Staphylococcus aureus* ATCC 25923) was determined.

### 2.4. Characterization of the Antibacterial Peptide

#### 2.4.1. Thermal Stability of the Antibacterial Peptide

To determine the thermal stability of the extracted antibacterial peptide from *S. cerevisiae,* 5 mL (10 mg/mL) of the active peptide was heated at 50, 60, 70, 80, 90, 100, 110, and 120 °C for 30 min. The antibacterial activity was estimated by the agar well diffusion agar activity against *E. coli* ATCC 25922 and *Staphylococcus aureus* ATCC 25923. Next, 100 μL of the extracted bioactive peptide was transferred to three wells in a Petri dish containing a Nutrient agar medium. The non-heated bioactive peptide was used as a control sample. The percentage of antibacterial activity was calculated using the following equation:

Inhibitory activity (%) = (Ac − As/Ac) × 100

where Ac is the inhibition zone of control sample, and As is the inhibition zone of test sample.

#### 2.4.2. pH Stability of the Antibacterial Peptide

The lyophilized purified antibacterial peptide from *S. cerevisiae* was dissolved in distilled water at 10 mg/mL and adjusted with 1N NaOH or 1N HCl to different pH values of 2, 3, 4, 5, 6, 7, 8, 9, and 10. After incubation for 24 h at 4 °C and 25 °C, the solutions containing the samples were adjusted to pH 7.0 ± 0.02 with a 0.5 M sodium citrate buffer. The inhibition activity of the peptide was then determined using the agar well diffusion agar method against *E. coli* ATCC 25922 and *Staphylococcus aureus* ATCC 25923. After incubation for 24 h at 37 °C, the percentage of antibacterial activity was calculated [19].

#### 2.4.3. Molecular Weight of the Antibacterial Peptide

The molecular weights of the antibacterial peptide extracted from *S. cerevisiae* were analyzed using sodium dodecyl sulphate with 15% polyacrylamide gel electrophoresis (SDS-PAGE) as described by Judd [20]. Then, 1 mg/mL of the antibacterial peptide and standard proteins (α-Lactalbumin 14.4 kDa, Aprotinin 6.5 kDa, Glucagon 3.8 kDa, and Insulin-A 2.5 kDa) (Promega Company, Madison, WI, USA) were dissolved in a phosphate buffer and transferred to a vertical slab chamber (10 cm × 10 cm × 0.6 mm). The gel was run at 50 mA and 50–70V for 3 h. The molecular weight of the extracted antibacterial peptide was determined after the relative mobility (Rm) of the antibacterial peptide and marker proteins were determined per the following equation:

Rm = the traveled distance of the peptide or marker proteins/traveled distance of methylene blue dye

### 2.5. Mode of Action

The modes of action (bacteriostatic) of the extracted antibacterial peptide from *S. cerevisiae* were assayed as described by [10]. The 6 log cfu/mL cultures of four indicator strains were cultivated into 100 mL of Nutrient broth (Himedia, Mumbai, India). The lyophilized purified antibacterial peptide was added to the indicator strain cultures at a final concentration of 0.01% (*w:v*). The samples and the control sample (without antibacterial peptide) were incubated at 37 °C. Indicator strain suspensions were taken at 3, 6, 12, 18, and 24 h, and the absorbance was measured by spectrophotometry (Sunny UV.7804C, Tokyo, Japan) at OD_600_. The viable bacteria cells were determined on the nutrient agar at various incubation periods [12,21].

### 2.6. Statistical Analysis

Statistical analyses of the different treatments cited above were conducted using the SPSS Statistics V22.0 (Statistical Package for Social Sciences, San Antonio, TX, USA). An analysis of the variance (ANOVA table) of the data was conducted and means for treatment values were analyzed (*p* ≤ 0.05) with least significant difference (LSD). Differences were considered significant at *p* ≤ 0.05.

## 3. Results and Discussion

### 3.1. Optimum Conditions of for Antibacterial Peptides Production

Both gram-negative bacteria tested including *E. coli* and *Klebsiella aerogenes* were inhibited by the presence of the antibacterial peptides produced from *Saccharomyces cerevisiae* in all the conditions tested (at the four time points and four different temperatures). This was not the case when testing the other two gram-positive bacteria including *Bacillus subtilis* and *Staphylococcus aureus,* which better tolerated the presence of the antibacterial peptides produced from *Saccharomyces cerevisiae.* This could be explained in part by the presence of cell walls comprised of thick layers of peptidoglycan in the case of Gram-positive bacteria. However, Gram-negative bacteria are known to have cell walls with a thin layer of peptidoglycan. The optimum conditions for the production of active peptides from yeast were 25–30 °C for 48 h, resulting in the highest inhibition towards *E. coli* and *Klebsiella aerogenes*. At 20 °C and 35 °C, the detected antibacterial activity was negligible with no effect against the four indicator strains (*Bacillus subtilis*, *E. coli*, *Klebsiella aerogenes*, and *Staphylococcus aureus*) especially at 24, 72, and 96 h (Table 1). 

### 3.2. Purification of the Antibacterial Peptide

**Figure 1:**
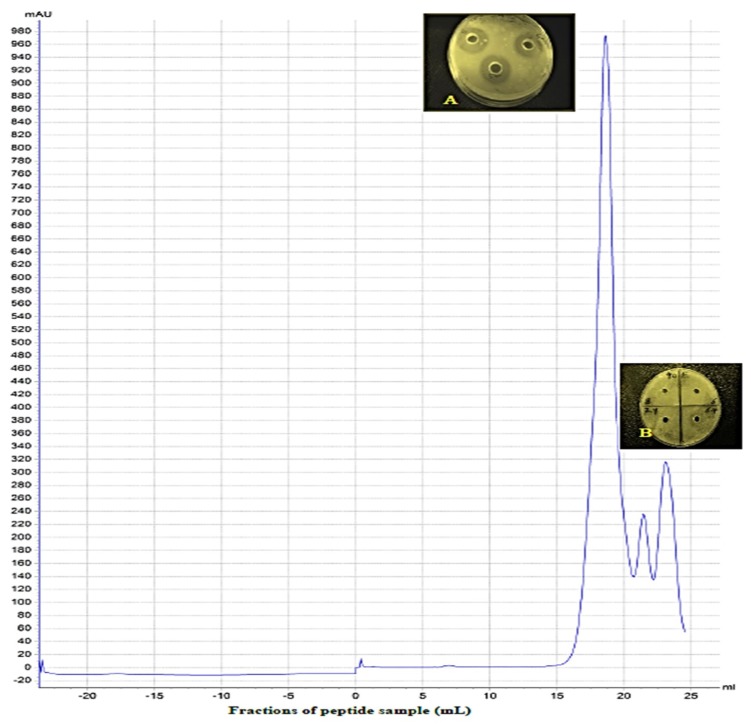
Chromatogram of gel filtration for antibacterial peptides from *Saccharomyces cerevisiae* by ÄKTA Pure 25 using Superdex 200 10/300 GL. (**A**) Inhibition zones of *E. coli* by peak1, (**B**) non-inhibition zone of *E. coli* by peak2 and peak3.

The extracted yeast peptides were passed through super filtration membranes of 10 kDa and 2 kDa and then freeze-dried. For further purification of the antibacterial peptide fractions, an ÄKTA purifier system was employed. Three peaks appeared after the ÄKTA Pure treatment. Fractions of 21–22 mL (peak 2) and 23–25 mL (peak 3) did not show growth inhibition activity against the two tested bacterial strains, while fractions of 16–20 mL (peak 1) showed the highest growth inhibition (Figure 1). The yield of the antibacterial peptide was 155 mg/100 mL of the culture medium. The inhibition zones were 24 and 20 mm for *E. coli* ATCC 25922 and *Staphylococcus aureus* ATCC 25923, respectively. Gram-negative bacteria (*E. coli*) appeared to be more sensitive to antibacterial peptide fractions than gram-positive (*S. aureus*) bacteria. These results were in agreement with Fakruddin et al. [13] who reported that Gram-negative bacteria were more sensitive to yeast peptides when compared to gram-positive bacteria. The inhibition activity of peptides increased proportionally with the α-helix of hydrophobic C-terminal peptides. This feature may be related to the composition of the amphipathic amino acids that are necessary for binding the bacterial cell membranes [19,22].

### 3.3. Characterization of the Antibacterial Peptide

#### 3.3.1. Thermal Stability of the Antibacterial Peptide

**Figure 2:**
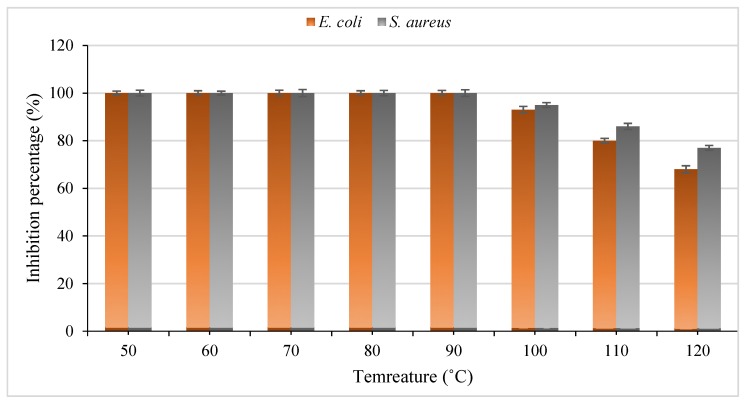
The thermal stability of the antibacterial peptide production from *Saccharomyces cerevisiae*.

The effect of temperature on the *S. cerevisiae* antibacterial peptide was determined (Figure 2). The results showed that the antibacterial peptide from yeast was stable at different temperatures ranging from 50 to 90 °C during 30 min of treatment. Interestingly, an antibacterial activity of 93% and 95% for *E. coli* and *S. aureus* was sustained even at 100 °C during 30 min of treatment. After 120 °C for 30 min, the antibacterial activity of the peptide was 68% and 77% for *E. coli* and *S. aureus*, respectively. This thermostable property makes this antibacterial peptide suitable for use in sterilization and thermal processes. The thermal stability of this small peptide could be related to the nature and chemical structures of such peptides, including the primary protein’s structure and with low molecular weight. Similar reports have shown that antibacterial peptides isolated from different microbial sources are able to persist at high temperatures without any change in the antimicrobial activity [23,24].

#### 3.3.2. Effect of pH on the Antibacterial Peptide

**Figure 3:**
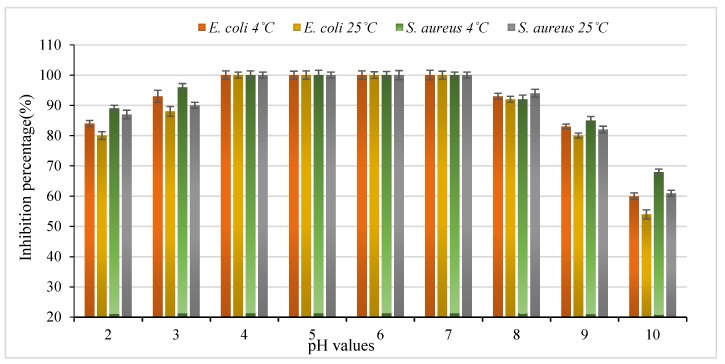
Stability of antibacterial peptide production from *Saccharomyces cerevisiae* under different pH and temperature conditions.

In order to address the pH properties including dependent and independent effects, the above fragments were estimated for antibacterial activity inhibition against two indicator strains; *E. coli* ATCC 25922 and *Staphylococcus aureus* ATCC 25923. The effects of the pH values on the stability of the antibacterial peptide from *S. cerevisiae* are shown in Figure 3. An evaluation of the pH value stability revealed that the antibacterial peptide remained stable after 24 h at 4 °C and 25 °C at pH values ranging from 4.0 to 7.0.

The inhibition activity of the peptide with extreme pH values 2, 3, 8, 9, and 10 against bacterial strains was also tested (Figure 3). Similar properties were previously studied for active peptides produced from yeasts, which were easily inactivated by strong acidic and alkaline conditions in different media. These characteristics limit the usage of peptides in food production with acidic and alkaline food. The antibacterial activity of this peptide was evaluated at different pH values at two temperatures (4 °C and 25 °C). The produced peptide was more effective at 4 °C within a pH range from 4 to 7 against the bacterial strains tested when compared to 25 °C. There was no significant difference (p > 0.05) between samples of *S. aureus* when tested at 4 °C and 25 °C at pH value 2, while there was a significant difference (*p* < 0.05) between samples of *E. coli* tested under the same conditions. In contrast, a significant difference (*p* < 0.05) between samples of *S. aureus* and *E. coli* tested at 4 °C and 25 °C at a pH value of 2 was observed. Furthermore, the analysis showed the presence of significant differences (*p* < 0.05) between samples of *S. aureus* tested at 4 °C and 25 °C at pH value 3. We also observed a significant difference (*p* < 0.05) between samples of *E. coli* when tested at 4 °C and 25 °C at pH 3. When comparing the two bacterial strains, we observed the absence of any significant difference (*p* > 0.05) between samples of *S. aureus* and *E. coli* tested at 4°C and pH 3. The presence of significant differences (*p* < 0.05) between samples of *S. aureus* tested at 4°C at pH 10 were also observed. However, no significant differences (*p* > 0.05) between samples of *S. aureus* tested at 25 °C and *E. coli* tested at 4 °C at pH value of 10 were observed. Interestingly, the lowest inhibition (%) value was observed in *E. coli* when tested at 25 °C and at pH 10.

Overall, the newly discovered produced peptide possessed high thermal stability and a wide range of pH stability when compared to other peptides produced from microorganisms (e.g., bacteriocins) as described in previous studies [25,26]. Moreover, antibacterial peptides acting in neutral and acidic environments are expected to provide protection from many unacceptable microorganisms that grow in these environments, contaminating food and causing spoilage [27].

#### 3.3.3. Molecular Weight of the Peptide

**Figure 4:**
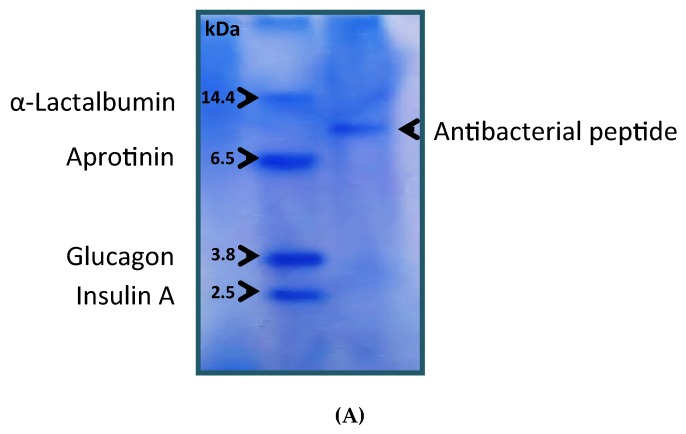

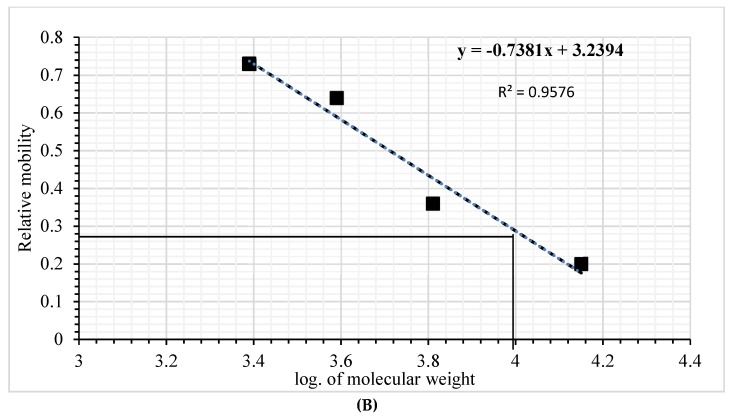
The molecular weight of antibacterial peptide production from *Saccharomyces cerevisiae* was determined by electrophoresis method. (**A**) Sodium dodecylsulfate polyacrylamide gel electrophoresis of standard proteins and the antibacterial peptide. (**B**) Relative mobility of standard proteins and the antibacterial peptide.

The purified antibacterial peptide from active fractions (peak1) was analyzed using SDS-PAGE and showed a single band. The molecular weight of the antibacterial peptide, as determined by relative mobility, was approximately 9770 Da (Figure 4), which is similar to the small molecular weight of other isolated peptides from *Klebsiella pneumonia,* as described in previous studies [28]. Thus, our results strongly suggest that peak 1 (9770Da) correspond to this peptide and might match the antibacterial activity produced by *S. cerevisiae* and thus be responsible for the bioactive activity shown against the different bacterial strains tested. In this sense, to better understand the role of molecular weight distribution in the inhibition of antibacterial activity of brewer’s yeast, a fraction analysis using ultrafiltration with 10 kDa cutoff membranes was performed. The results showed that 3–10 kDa fractions were fundamentally comprised of smaller peptides with biological activity, which is in agreement with past studies that indicated that antibacterial peptides are small peptides [9].

### 3.4. Mode of Action

**Figure 5:**
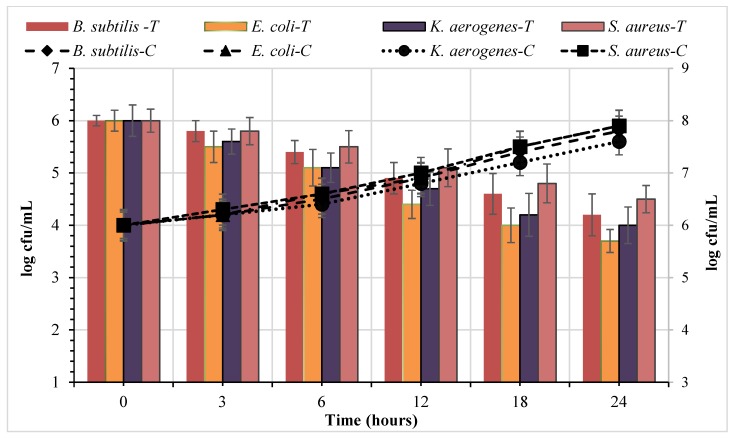
The mode of action of the antibacterial peptide from *Saccharomyces cerevisiae* against the four indicator bacteria strains.

The antibacterial peptide effect of *S. cerevisiae* on cell viability (kill time) from four indicator strains is shown in Figure 5. The antibacterial peptide reduced the viability of target bacteria compared to the control sample. The antibacterial peptide’s efficacy depended on both the concentration of added peptide and exposure time. Additionally, the movement of small peptide and their spread during agar well diffusion appeared 3 h following the addition of the antibacterial peptide with 5.8, 5.5, 5.6, and 5.8 log (cfu/mL) reduction of *B. subtilis*, *E. coli*, *K. aerogenes,* and *S. aureus*, respectively. The statistical analysis of log (cfu/mL) reduction for each bacteria strain was estimated at interval times of 3, 6, 12, 18, and 24 h. The result showed that the antibacterial peptide effect on decreasing the viable cell counts was significant (*p* < 0.05) between the control samples and all four bacterial strains among the five interval times tested. In contrast, there was no significant difference (*p* > 0.05) between either Gram-positive strains or Gram-negative strains. In addition, the peptide’s inhibition effect on Gram-negative strains was highly significant (*p* > 0.05) when compared with Gram-positive strains. After 24 h of incubation, the peptide’s inhibition effect on Gram-negative strains was high compared with that of Gram-positive strains. The antibacterial peptide caused a decrease in the viable cell counts of gram-negative strains ranging from 2 to 2.3 log. units along the evaluated times in comparison to gram-positive strains which ranged from 1.5 to 1.8 log. units. The kill-time of this peptide was in agreement with that from earlier reports [29,30]. The mode of action of antimicrobial peptides fundamentally depends on the reaction of bioactive peptides with the membrane of bacteria cells and the cells’ internal composition [31]. Generally, the antimicrobial peptides were effective due to the electrostatic reaction with the cell membranes. In order to understand the mechanism behind antimicrobial peptides, the results of various methods have suggested that adsorption of bioactive peptides will occur on the bacterial cell membrane, leading to complete damage to the membrane. For example, the brave straw model, aggregate model, carpet model, and toroid pore model are critically considered models of bioactive peptides as antibacterial compounds [27,32].

## 4. Conclusions

*S. cerevisiae* belongs to the eukaryotic kingdom is nonpathogenic, and due to its long history of use in the production of consumable products such as ethanol, many baked products, and pastries, it has been classified as a generally regarded as safe organism (GRAS). In this study, antibacterial peptide was produced and isolated from *Saccharomyces cerevisiae* (Baker’s yeast) by an ultrafiltration process (two membranes with cut-offs 2 and 10 kDa) and purified using the ÄKTA Pure 25 system. The antibacterial peptide activity was then characterized and studied against four bacterial strains. The results showed that the peptide produced by *Saccharomyces cerevisiae* had a molecular weight of 9.77 kDa and exerted inhibition activity against both Gram-negative and Gram-positive bacteria. The produced peptide was more effective at 4 °C within a pH range of 4-7 against the bacterial strains tested when compared to 25 °C, while the lowest inhibition (%) was observed in *E. coli* when tested at 25 °C and at a pH value of 10. In addition, the peptide was thermostable and steady with pH values ranging from 4–7, which is a very important aspect in food production for sterilization and thermal processes. The isolated antibacterial peptide demonstrated its potential as a bio-preservative in food manufacturing. Although the current study isolated a peptide containing a novel bioactive compound from *Saccharomyces cerevisiae*, additional research regarding the amino acid sequence and structure of this antibacterial peptide is warranted.

## Figures and Tables

**Table 1:** The optimum conditions for antibacterial peptides produced from Saccharomyces *cerevisiae.*

Strains	24 h				48 h				72 h				96 h			
	20 °C	25 °C	30 °C	35 °C	20 °C	25 °C	30 °C	35 °C	20 °C	25 °C	30 °C	35 °C	20 °C	25 °C	30 °C	35 °C
*Bacillus subtilis* ATCC 23857	-	-	+	+	+	+	++	++	-	-	+	+	-	-	+	-
*Escherichia coli* ATCC 25922	+	+	++	+	++	+++	+++	++	+	+	++	+	+	+	+	-
*Klebsiella aerogenes* ATCC 13048	+	+	++	++	++	+++	+++	+++	+	++	++	++	+	+	++	+
*Staphylococcus aureus* ATCC 25923	-	-	+	+	+	+	++	+	-	-	+	-	-	-	+	-

Diameter of inhibition zone (mm): +++: 16–20; ++: 12–16; +: 8–12; −:no inhibitory activity (including the 6mm diameter of each well).
